# Multi-Agent DDPG-Based Multi-Device Charging Scheduling for IIoT Smart Grids

**DOI:** 10.3390/s25175226

**Published:** 2025-08-22

**Authors:** Haiyong Zeng, Yuanyan Huang, Kaijie Zhan, Zichao Yu, Hongyan Zhu, Fangyan Li

**Affiliations:** 1 Guangxi Key Laboratory of Braininspired Computing and Intelligent Chips, School of Electronic and Information Engineering/School of Integrated Circuits, Guangxi Normal University, Guilin 541001, China; zenghaiyong@stu.hit.edu.cn (H.Z.); yyy_59o3o@163.com (Y.H.); kaijiezhan@foxmail.com (K.Z.); 2 School of Electronic and Information Engineering, Harbin Institute of Technology, Shenzhen 150001, China; 3 Department of Electronic Engineering and Information Science, University of Science and Technology of China, Hefei 230026, China

**Keywords:** electric vehicle charging scheduling, deep reinforcement learning, cost optimization management, smart grids, smart sensors, industrial internet of things

## Abstract

As electric vehicles (EVs) gain widespread adoption in industrial environments supported by Industrial Internet of Things (IIoT) smart grids technology, coordinated charging of multiple EVs has become vital for maintaining grid stability. In response to the scalability challenges faced by traditional algorithms in multi-device environments and the limitations of discrete action spaces in continuous control scenarios, this paper proposes a dynamic charging scheduling algorithm for EVs based on Multi-Agent Deep Deterministic Policy Gradient (MADDPG). The algorithm combines real-time electricity prices, battery status monitoring, and distributed sensor data to dynamically optimize charging and discharging strategies of multiple EVs in continuous action spaces. The goal is to reduce charging costs and balance grid load through coordinated multi-agent learning. Experimental results show that, compared with baseline methods, the proposed MADDPG algorithm achieves a 41.12% cost reduction over a 30-day evaluation period. Additionally, it effectively adapts to price fluctuations and user demand changes through Vehicle-to-Grid technology, optimizing charging time allocation and enhancing grid stability.

## 1. Introduction

With increasing global environmental awareness, electric vehicles (EVs) are experiencing significant growth worldwide. EVs encompass not only traditional transportation vehicles such as electric cars and trucks, but also industrial equipment, including electric forklifts and self-driven vehicles. These battery-powered devices provide solutions for reducing carbon emissions and decreasing reliance on fossil fuels. As technology advances, EVs have become more common in industrial sectors, creating opportunities for coordinated energy management [1,2,3,4]. However, the widespread adoption of EVs introduces challenges for power grid management, particularly concerning dynamic charging demands and load variations. The charging requirements must account for real-time grid load conditions and involve intelligent adjustments to prevent excessive strain during peak demand periods [5]. Consequently, the development of efficient EV charging scheduling methods has become important for ensuring grid stability [6].

The advancement of smart grid technologies has transformed energy management systems through intelligent sensing and communication capabilities. Modern charging infrastructure leverages smart sensors to monitor critical parameters, including battery state-of-charge, grid conditions, and dynamic electricity pricing in real-time [7,8,9]. The integration of Industrial Internet of Things (IIoT) [10,11,12] further enhances fleet management through predictive frameworks and real-time data exchange [13], while simultaneously raising cybersecurity concerns regarding the protection of sensitive charging data. Within this interconnected environment, the main challenge becomes coordinated decision-making in continuous action spaces, where each EV’s charging actions directly affect overall system performance. This IIoT-enabled infrastructure facilitates such coordination through continuous data exchange, enabling accurate system monitoring and improved energy management.

Traditional optimization methods have demonstrated value in early studies but reveal significant limitations when confronted with multi-agent coordination challenges. Swarm intelligence algorithms such as genetic algorithms and particle swarm optimization [14,15] perform well in static scenarios but struggle with real-time adaptability and inter-agent coordination. Model predictive control [16] and Lyapunov optimization approaches [17] have shown promise in dynamic scenarios but face computational scalability issues when managing multiple autonomous agents with continuous action spaces. Additionally, these methods fail to address the fundamental challenge of non-stationary environments that arise when multiple learning agents operate simultaneously.

To address these challenges, Deep Reinforcement Learning (DRL) [18] has emerged as a breakthrough solution due to its ability to handle high-dimensional state spaces, continuous action spaces, and dynamic environments. However, most existing DRL applications in EV charging focus on single-agent scenarios, limiting their applicability to real-world multi-device environments. Single-agent Deep Q-Network (DQN) approaches [19], while effective for individual EV optimization, suffer from the curse of dimensionality when extended to multi-agent settings and cannot handle continuous charging power control effectively. To overcome the discrete action limitations of DQN, the Deep Deterministic Policy Gradient (DDPG) algorithm has been adopted for continuous EV charging control [20], demonstrating superior performance in individual EV optimization scenarios. However, these single-agent approaches fail to consider the collective performance and coordination requirements of multiple EVs operating simultaneously.

As mentioned above, the limitations of single-agent approaches become evident when managing multiple charging piles at charging stations. If all charging piles make decisions independently, the total power demand may exceed the transformer’s rated capacity, potentially affecting power system stability. Recent studies have addressed this by imposing constraints on total charging rates [21] and developing recurrent DDPG algorithms [22]. However, these approaches still rely on single-agent frameworks, which face significant scalability challenges in complex multi-device scenarios. Recognizing these limitations, multi-agent reinforcement learning (MARL) has gained attention for coordinating multiple decision-making units. MARL algorithms are primarily categorized into Q-learning-based methods, such as QMIX [23], and policy gradient-based methods, including Multi-Agent Deep Deterministic Policy Gradient (MADDPG). Recent advances in policy-gradient MARL have demonstrated superior capabilities in continuous control domains, with Actor-Critic methods enabling more precise control over EV charging and discharging behaviors [24]. These methods have shown particular effectiveness in industrial environments, where multi-agent systems can flexibly adapt to dynamic resource allocation challenges in terminal–edge–cloud IIoT architectures [25]. MADDPG has been specifically applied to charging decision problems involving multiple charging piles [26,27,28]. While MARL shows promise for EV charging coordination, some limitations remain in existing approaches [29,30]. Most existing methods employ limited inter-agent interaction or operate as independent single-agent systems, resulting in uncoordinated charging behaviors that may cause grid load imbalances or overload conditions when multiple EVs simultaneously access limited grid resources during peak demand periods.

Additionally, current charging coordination frameworks lack design considerations for Industrial IoT scenarios and fail to exploit the real-time data capabilities inherent in IIoT environments. This limitation prevents effective utilization of real-time grid conditions, dynamic pricing information, and EV status updates for optimized charging scheduling in industrial park applications. To address these limitations and research gaps, this paper proposes an MADDPG-based coordinated charging scheduling algorithm that integrates continuous action control with multi-agent coordination in IIoT-enabled environments.

The primary contributions of this paper are summarized as follows:

1. We propose an MADDPG-based coordination framework for smart sensor-integrated Industrial IoT environments that combines real-time multi-device sensor data with multi-agent reinforcement learning for EV charging scheduling. Our framework exploits smart sensor infrastructure characteristics, real-time data collection capabilities, and distributed communication protocols to enhance coordination performance and reduce charging costs in industrial park settings.
2. We propose an MADDPG-based multi-agent algorithm that facilitates coordinated policy learning among multiple EVs, ensuring continuous control over charging power. This approach allows each EV agent to autonomously decide based on local observations, effectively accommodating the diverse battery capacities and charging requirements of heterogeneous EVs. By utilizing continuous control, our algorithm overcomes the discrete action limitations found in traditional approaches like QMIX.
3. Comprehensive experimental evaluation demonstrates the effectiveness of the proposed sensor-integrated approach, achieving 43.5% cost reduction compared with baseline methods over a 30-day evaluation period while maintaining grid stability and satisfying each EV’s charging requirements under realistic industrial park scenarios with dynamic pricing, real-time sensor monitoring, and varying EV fleet sizes.

The rest of this paper is organized as follows. Section 2 presents the system model and problem formulation for multi-agent EV charging coordination. The design and implementation of the proposed MADDPG-based multi-device scheduling algorithm are discussed in Section 3. Section 4 provides the experimental setup and results, including performance evaluation against existing approaches. Finally, conclusions and future research directions are discussed in Section 5.

## 2. System Model and Problem Formulation

### 2.1. System Model

**Figure 1:**
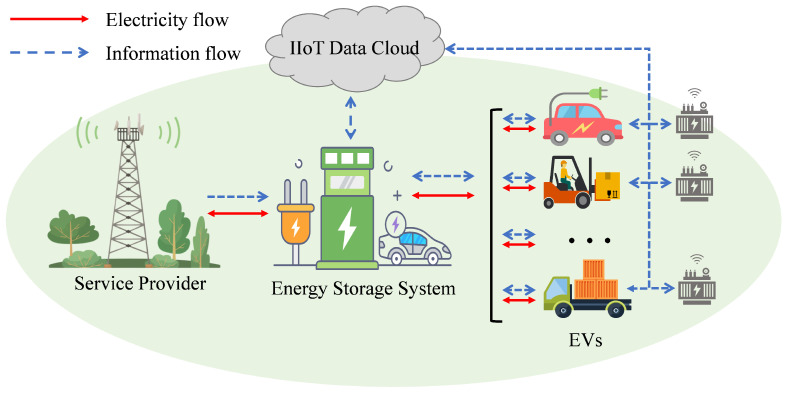
Multi-device charging scheduling system for IIoT smart grids.

We consider an industrial park equipped with *N* heterogeneous EVs, each requiring coordinated charging scheduling to optimize grid load profiles and minimize operational costs. Figure 1 illustrates the overall system architecture, where multiple EVs are connected to a centralized charging management system through IIoT infrastructure, enabling real-time data exchange and coordinated decision-making.

Each EV is connected to a bidirectional charging infrastructure that supports Vehicle-to-Grid (V2G) functionality, enabling both energy consumption from the grid and energy feedback during peak load periods. The IIoT infrastructure consists of three integrated components: embedded sensors on each EV that continuously monitor battery State of Charge (SoC), remaining charging duration before departure, and real-time charging status; smart charging stations equipped with bidirectional power converters and communication modules that interface with grid management systems to acquire electricity price signals and grid load conditions; and a centralized data aggregation unit that processes information for coordinated decision-making.

### 2.2. Battery State Model

The battery status of each EV is characterized by its SoC, representing the ratio of remaining energy to nominal battery capacity. The SoC evolves over discrete time intervals *t* based on applied charging or discharging power, with continuous monitoring through embedded sensor systems ensuring accurate state estimation.

For charging operations, the SoC update is

(1) $$SoC_{i,t+1}=SoC_{i,t}+\frac{\eta_{c}\cdot P_{i,t}^{ch}\cdot \Delta t}{C_{i}},$$

where $SoC_{i,t}$ is the SoC of EV *i* in time slot *t*, $P_{i,t}^{ch}\geq 0$ is the charging power, $C_{i}$ is the battery capacity, $\eta_{c}\in \left(0,1\right]$ is the charging efficiency, and Δt is the time slot duration.

For discharging operations, the SoC evolves as

(2) $$SoC_{i,t+1}=SoC_{i,t}-\frac{P_{i,t}^{dis}\cdot \Delta t}{\eta_{d}\cdot C_{i}},$$

where $P_{i,t}^{dis}\geq 0$ is the discharging power, and $\eta_{d}\in \left(0,1\right]$ is the discharging efficiency.

The SoC is constrained within feasible bounds:

(3) $$SoC_{\min}\leq SoC_{i,t}\leq SoC_{\max},\;\forall i,t,$$

Individual power limits are enforced:

(4) $$0\leq P_{i,t}^{ch}\leq P_{i,\max}^{ch},\;0\leq P_{i,t}^{dis}\leq P_{i,\max}^{dis},\;\forall i,t,$$

To prevent grid overload, the aggregate charging power is constrained:

(5) $$\sum_{i=1}^{N}P_{i,t}^{ch}\leq P_{\max},\;\forall t,$$

where $P_{\max}$ is the maximum permissible total charging power.

These constraints ensure safe battery operation and grid stability while enabling effective coordination among multiple charging devices through real-time monitoring and control.

### 2.3. Problem Formulation

The objective of the multi-device charging scheduling problem is to minimize the total electricity cost for all EVs over a finite time horizon *T* while satisfying individual charging requirements and grid constraints. The total cost for all EVs is defined as

(6) $$C=\sum_{t=1}^{T}\sum_{i=1}^{N}\left(P_{t}P_{i,t}^{ch}-P_{t}P_{i,t}^{dis}\right),$$

where $P_{t}$ is the time-varying electricity price at time slot *t*. Here, $P_{t}P_{i,t}^{ch}$ represents the cost incurred when purchasing electricity from the grid, while $P_{t}P_{i,t}^{dis}$ represents the revenue generated by selling energy back to the grid.

The optimization problem is formulated as

(7) $$\min_{P_{i,t}^{ch},P_{i,t}^{dis}}C$$

subject to the battery dynamics in (1) and (2), the constraints in (3)–(5), and the charging completion requirement:

(8) $$SoC_{i,t}\geq SoC_{target},\;\forall i,$$

This problem is challenging due to the coupled constraints across multiple agents, the continuous decision variables, and the stochastic nature of electricity prices, EV arrival/departure times, and initial SoC levels. Traditional optimization methods struggle with scalability and real-time adaptation in such dynamic multi-device environments. To address these issues, we model the problem as a multi-agent Markov Decision Process (MDP) and solve it using an MADDPG-based approach, as detailed in the next section.

## 3. Charging Scheduling Algorithm Based on MADDPG

The multi-agent EV charging coordination problem involves multiple autonomous agents operating in continuous action spaces with complex inter-agent dependencies. Traditional discrete algorithms such as DQN and Multi-Agent DQN face scalability challenges due to exponential growth in joint action spaces and discrete action limitations. The integration of real-time sensor data and dynamic system conditions further requires algorithms capable of processing high-dimensional observations and adapting to rapidly changing environments.

To address these challenges, we adopt the MADDPG algorithm, which combines centralized training with decentralized execution. MADDPG enables each agent to maintain its own actor network for continuous action selection based on local sensor observations, while employing centralized critics for value estimation using global state information. This design overcomes non-stationarity and high dimensionality challenges while preserving decentralized execution capabilities essential for real-time industrial operation.

### 3.1. Multi-Agent MDP Formulation

To implement the MADDPG framework for sensor-integrated EV charging coordination, we formalize the problem as a multi-agent Markov Decision Process. The multi-agent EV charging scheduling problem is modeled as an MDP defined by the tuple $\left(S,A,P,{\left\{r_{i}\right\}}_{i=1}^{N},\gamma \right)$, where:

(1) State $S_{t}$: At time slot *t*, the joint state $S_{t}\in S$ captures essential information of the environment and all EVs:

(9) $$S_{t}=\left[P_{t},SoC_{1,t},SoC_{2,t},\ldots ,SoC_{N,t},L_{t},T_{1,t}^{rem},\ldots ,T_{N,t}^{rem}\right],$$

where $P_{t}$ is the electricity price, $SoC_{i,t}$ is the State of Charge of the *i*-th EV battery, $L_{t}$ denotes the current grid load, and $T_{i,t}^{rem}$ represents the remaining charging time for EV *i*.

(2) Action $a_{t}$$=(a_{1,t},a_{2,t},\ldots ,a_{N,t})$: Each agent *i* chooses a continuous action $a_{i,t}\in \left[P_{i}^{min},P_{i}^{max}\right]$ representing its charging (positive) or discharging (negative) power at time *t*. The joint action $a_{t}$ must satisfy the aggregate charging power constraint:

(10) $$\sum_{i=1}^{N}\max\left(a_{i,t},0\right)\leq P_{\max}.$$

(3) State Transition Probability P: The environment dynamics are governed by unknown transition probabilities $P(S_{t+1}|S_{t},a_{t})$, capturing the evolution of EV battery states and grid status following joint actions. Due to system complexity and stochasticity, these dynamics are learned implicitly via interaction without explicit modeling.

(4) Reward $r_{i,t}$: Each agent receives a reward designed to minimize electricity costs, ensure battery safety, encourage timely charging completion, and maintain grid stability. The reward function is formulated as

(11) $$r_{i,t}=-P_{t}\cdot a_{i,t}+R_{lim}^{i,t}+R_{target}^{i,t}$$

where the constraint penalty $R_{lim}^{i,t}$ and target completion reward $R_{target}^{i,t}$ are defined as

(12) $$R_{lim}^{i,t}=\left\{\begin{array}{cc} -\rho_{1}, & \mathrm{if}\;SoC_{i,t}<SoC_{\min}\;\mathrm{or}\;SoC_{i,t}>SoC_{\max}\\ -\rho_{2}, & \mathrm{if}\;\sum_{i=1}^{N}\max\left(a_{i,t},0\right)>P_{\max}\\ 0, & \mathrm{otherwise} \end{array}\right.$$

(13) $$R_{target}^{i,t}=\left\{\begin{array}{cc} -\rho_{3}, & \mathrm{if}\;T_{i,t}^{rem}=0\;\mathrm{and}\;SoC_{i,t}<SoC_{target}\\ 0, & \mathrm{otherwise} \end{array}\right.$$

(5) Discount factor γ∈(0,1]: Balances the importance of immediate and future rewards to encourage policies optimizing long-term performance.

### 3.2. MADDPG Algorithm Implementation

To address the continuous action space and multi-agent coordination challenges in EV charging scheduling, we implement the MADDPG algorithm, which extends the deterministic policy gradient framework to multi-agent contexts through centralized training with decentralized execution.

Each EV agent *i* employs an actor network $\mu_{\theta_{i}}$ that maps its local observation $o_{i}$ to a continuous action $a_{i}=\mu_{\theta_{i}}\left(o_{i}\right)$. During training, each agent utilizes a centralized critic network $Q_{\phi_{i}}\left(s,a\right)$ that processes the global state *s* and joint action $a=(a_{1},a_{2},\ldots ,a_{N})$ to address multi-agent non-stationarity, while maintaining decentralized actor execution based solely on local observations.

The critic network parameters $\phi_{i}$ are optimized by minimizing the temporal difference loss:

(14) $$L\left(\phi_{i}\right)=E_{\left(s,a,r_{i},s^{\prime }\right)}\left[{\left(Q_{\phi_{i}}\left(s,a\right)-y_{i}\right)}^{2}\right],$$

where the target value $y_{i}$ is computed using target networks:

(15) $$y_{i}=r_{i}+\gamma Q_{\phi_{i}^{-}}\left(s^{\prime },a_{1}^{\prime },\ldots ,a_{N}^{\prime }\right),$$

with $a_{j}^{\prime }=\mu_{\theta_{j}^{-}}\left(o_{j}^{\prime }\right)$ representing the next action from the target actor network.

The actor network parameters $\theta_{i}$ are updated using the deterministic policy gradient:

(16) $$\nabla_{\theta_{i}}J\approx E_{s}\left[\nabla_{\theta_{i}}\mu_{\theta_{i}}\left(o_{i}\right)\nabla_{a_{i}}Q_{\phi_{i}}\left(s,a_{1},\ldots ,a_{N}\right){|}_{a_{i}=\mu_{\theta_{i}}\left(o_{i}\right)}\right].$$

Training stability is enhanced through experience replay and soft target network updates:

(17) $$\theta_{i}^{-}\leftarrow \tau \theta_{i}+\left(1-\tau \right)\theta_{i}^{-},\;\phi_{i}^{-}\leftarrow \tau \phi_{i}+\left(1-\tau \right)\phi_{i}^{-},$$

where τ≪1 controls the update rate.

**Figure 2:**
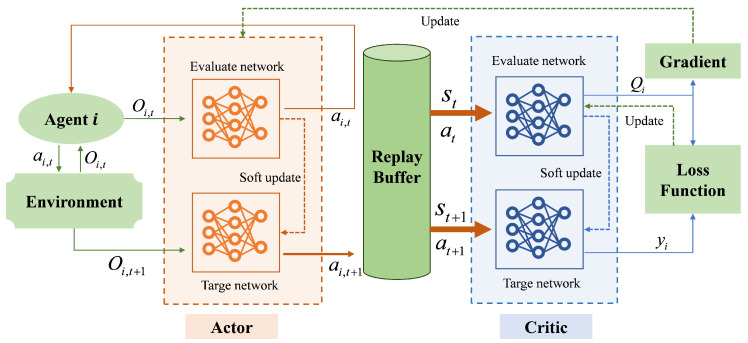
MADDPG-based charging scheduling framework: decentralized actors generate continuous charging/discharging actions from local observations, while centralized critics evaluate joint state-action pairs for coordinated training.

Algorithm 1 summarizes the training process for coordinated EV charging scheduling with real-time sensor integration. The algorithm initializes all network parameters, then iteratively collects experience through environment interaction with continuous sensor monitoring and updates network parameters using sampled minibatches. The MADDPG framework effectively handles multi-agent coordination and continuous charging power control through its combination of decentralized actors and centralized critics, as illustrated in Figure 2.
**Algorithm 1** MADDPG-based Multi-Agent EV Charging Scheduling1:Initialize actor networks $\mu_{\theta_{i}}$, critic networks $Q_{\phi_{i}}$, and corresponding target networks $\mu_{\theta_{i}^{-}},Q_{\phi_{i}^{-}}$ for all agents i=1,…,N.2:Initialize experience replay buffer D.3:**for** episode = 1 to *M* **do**4:    Reset environment, obtain initial state $s=(o_{1},\ldots ,o_{N})$.5:    **for** time step t=1 to *T* **do**6:        **for** each agent *i* **do**7:           Select action $a_{i}=\mu_{\theta_{i}}\left(o_{i}\right)+N_{t}$, where $N_{t}$ is exploration noise.8:        **end for**9:        Execute joint action $a=(a_{1},\ldots ,a_{N})$, observe reward $r=(r_{1},\ldots ,r_{N})$, and next state $s^{\prime }=\left(o_{1}^{\prime },\ldots ,o_{N}^{\prime }\right)$.10:        Store transition $\left(s,a,r,s^{\prime }\right)$ into D.11:        **if** replay buffer size sufficient **then**12:           Sample minibatch from D.13:           **for** each agent *i* **do**14:               Update critic network $Q_{\phi_{i}}$ by minimizing loss $L(\phi_{i})$.15:               Update actor network $\mu_{\theta_{i}}$ using policy gradient.16:               Soft-update target networks:$$\theta_{i}^{-}\leftarrow \tau \theta_{i}+\left(1-\tau \right)\theta_{i}^{-},\;\phi_{i}^{-}\leftarrow \tau \phi_{i}+\left(1-\tau \right)\phi_{i}^{-}.$$17:           **end for**18:        **end if**19:        Update $s\leftarrow s^{\prime }$.20:    **end for**21:**end for**

### 3.3. Computational Complexity Analysis

The computational complexity of the proposed MADDPG-based charging scheduling depends on the neural network architectures and the number of agents. Consider actor networks with $L_{a}$ layers and $n_{a}$ neurons per hidden layer, and critic networks with $L_{c}$ layers and $n_{c}$ neurons per hidden layer. The input dimensions are $d_{o}$ for actors (local observation) and $d_{s}+N\times d_{a}$ for critics (global state plus joint actions), where *N* is the number of agents and $d_{a}$ is the action dimension.

The per-step complexity for each actor network is $O(d_{o}\cdot n_{a}+\left(L_{a}-2\right)n_{a}^{2}+n_{a}\cdot d_{a})$, while for each critic network, the complexity is $O(\left(d_{s}+Nd_{a}\right)\times n_{c}+\left(L_{c}-2\right)n_{c}^{2}+n_{c})$. The critic input dimension scaling with *N* introduces quadratic dependence on the number of agents, reflecting the centralized training overhead. The experience replay buffer requires memory complexity of $O(2d_{s}+N\left(d_{o}+d_{a}+1\right))$ per sample.

During execution, only decentralized actor networks are active, with inference complexity $O(N\times \left(d_{o}n_{a}+\left(L_{a}-2\right)n_{a}^{2}+n_{a}d_{a}\right))$ that scales linearly with the number of agents, enabling real-time operation. The MADDPG approach introduces computational overhead compared with single-agent methods due to centralized critics, but remains computationally feasible for moderate numbers of EV agents while effectively balancing complexity and coordination performance.

## 4. Experimental Results and Discussion

To validate the effectiveness of the proposed MADDPG-based charging scheduling algorithm, we construct a simulation environment modeling the charging behavior of electric vehicles and their interactions with the power grid in an industrial park. The simulation integrates factors including real-time electricity price data, battery state dynamics, and charging time management.

### 4.1. Setup and Training

The simulation environment utilizes hourly electricity price data sourced from the UK NordPool database [31]. The training utilizes six months of historical pricing data, while the evaluation employs completely separate monthly periods to ensure unbiased performance assessment and generalization capability. These six months of real-time electricity price data are stored in a table for dynamic retrieval during the training process to ensure experimental authenticity. The electricity price unit remains consistent with the source data, using British pounds (GBP) as the unit of measurement.

In the simulation environment, the charging and discharging behaviors of EVs are modeled with the following assumptions to simplify the problem:

1. EVs utilize lithium-ion batteries with constant charging and discharging power rates, with varying battery capacities and maximum charging power limits across different vehicles.
2. All EVs participate in charging and discharging processes within the industrial park using conventional slow-charging methods.
3. Charging and discharging decisions are influenced by dynamic electricity prices obtained through real-time pricing signals, without considering external disturbances or physical queuing effects at charging stations (This assumption is suitable for IIoT environments with sufficient charging infrastructure and centralized management, where external disturbances and queuing effects can be reasonably neglected [32,33]).
4. EVs commence charging immediately upon arrival at charging stations, with charging periods aligned to hourly intervals.
5. Battery safety is maintained by constraining SoC between 0.1 and 1.0, with continuous monitoring through simulated sensor feedback systems.

The simulation environment models five heterogeneous EVs to reflect realistic industrial park scenarios. Due to varying battery capacities across vehicles, SoC is adopted as a unified standard rather than assuming fixed capacities. The scheduling operates with one-hour time intervals, where each training episode corresponds to one complete operational day of 24 h divided into 24 time slots. EV behavioral parameters [34] are modeled using truncated normal distributions to enhance the algorithm’s generalization capability, and the algorithm does not rely on prior knowledge of stochastic variable distributions. Table 1 details the specific parameter settings for EV behavior modeling.

The core hyperparameters of the MADDPG training process are carefully configured to balance learning stability and convergence speed. These include the learning rate, discount factor, exploration noise parameters, replay buffer capacity, batch size, and the target networks’ soft update coefficient. The complete set of algorithm parameters used in training is summarized in Table 2. Additionally, the system configuration parameters are in Table 3.

**Figure 3:**
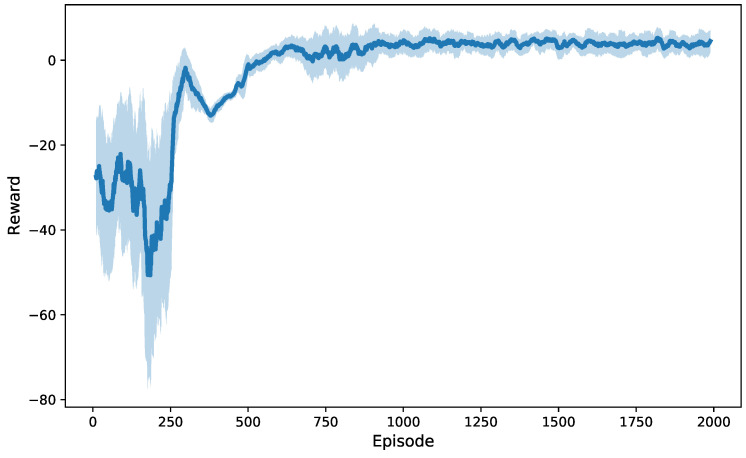
Reward variation graph during MADDPG training.

Figure 3 illustrates the training convergence of the proposed MADDPG algorithm across 20 independent training runs with different random seeds. The solid line represents the mean reward progression, while the shaded area indicates the 95% confidence interval. The initial training phase exhibits significant reward fluctuations due to random exploration as agents learn coordination strategies while simultaneously adapting to real-time data streams from multiple sensors. As training progresses, the reward fluctuations gradually diminish and converge toward higher values, indicating consistent learning of coordinated charging policies that effectively utilize real-time sensor feedback for decision-making across different initializations.

**Figure 4:**
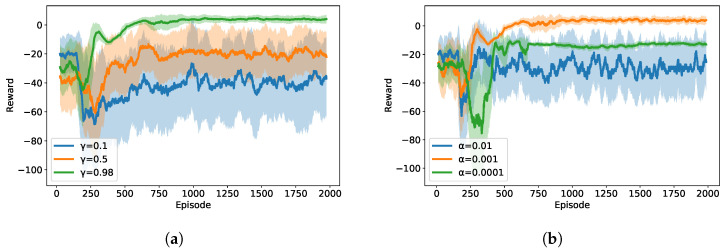
Parameter sensitivity analysis graph for MADDPG algorithm: (**a**) Effect of discount factor on MADDPG performance. (**b**) Effect of learning rate on MADDPG performance.

Figure 4 demonstrates the parameter sensitivity analysis for the proposed MADDPG algorithm. The optimal discount factor γ=0.98 achieves the best performance with rapid convergence, while lower values result in suboptimal coordination due to insufficient long-term planning. The learning rate $\alpha =\alpha_{a}=\alpha_{c}=0.001$ provides optimal balance between convergence speed and final performance quality, whereas higher rates cause training oscillations and lower rates converge quickly but fail to reach optimal reward values.

### 4.2. Performance Evaluation

**Figure 5:**
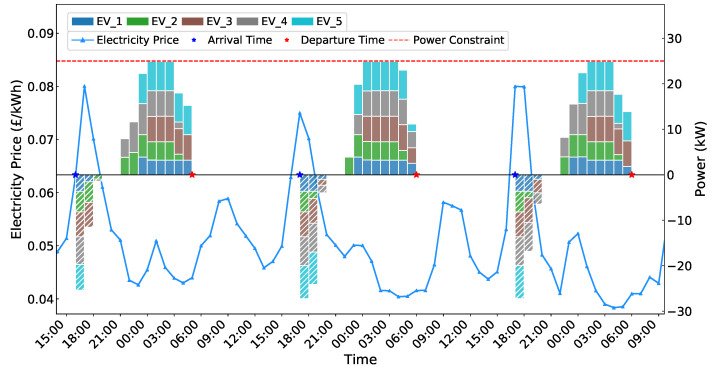
EVs’ charging and discharging behavior diagram.

**Figure 6:**
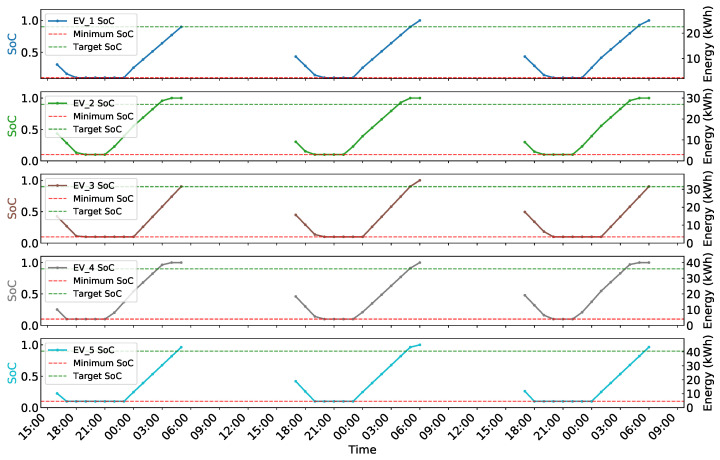
SoC variation graph of EVs.

Figure 5 and Figure 6 demonstrate the effectiveness of the proposed MADDPG-based coordinated charging scheduling algorithm in managing multi-agent EV charging operations. Figure 5 presents the charging and discharging behavior of five EVs over a three-day period under dynamic electricity pricing. The results show that EVs successfully identify electricity price variations and make corresponding charging or discharging decisions accordingly. The algorithm effectively schedules charging operations during low-price periods and discharging operations during high-price periods through V2G functionality. The charging behaviors respect individual power constraints without exceeding the maximum power limits, while the aggregate power consumption remains within the 25 kW system constraint.

Figure 6 illustrates the battery state evolution throughout the charging process. Despite having different battery capacities, all five vehicles successfully reach their target SoC levels before departure while maintaining safe operation within the constraint range. These results confirm the algorithm’s ability to achieve collective optimization while satisfying individual EV requirements.

**Figure 7:**
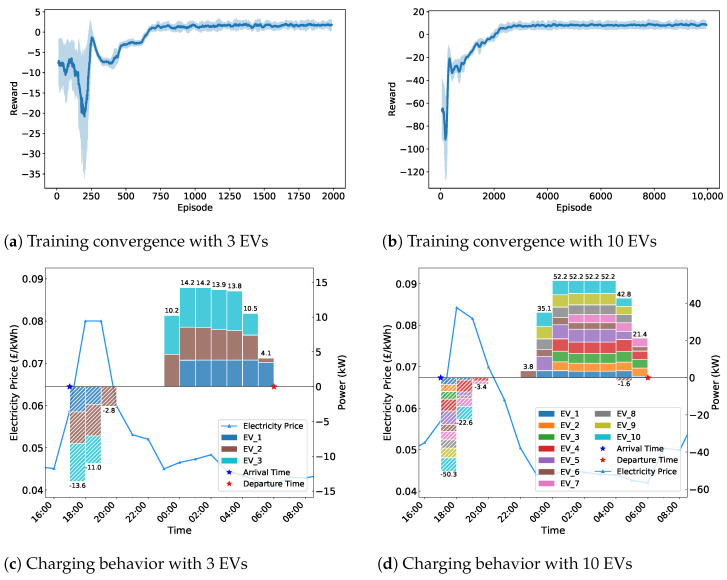
Scalability analysis of MADDPG algorithm under different EV fleet sizes: (**a**,**b**) Training convergence curves showing reward evolution during learning process. (**c**,**d**) Charging behavior patterns demonstrating coordinated scheduling strategies.

To evaluate the scalability of the proposed algorithm, we conducted experiments with different EV fleet sizes and power constraints. Figure 7 demonstrates the algorithm’s performance across different fleet sizes. The results show that the algorithm successfully converges for both 3 EV and 10 EV scenarios. The 3 EV case achieves faster convergence due to lower system complexity, while the 10 EV scenario requires more training episodes but eventually reaches stable performance. This validates that the MADDPG framework scales effectively to accommodate varying fleet sizes while preserving coordination quality.

**Figure 8:**
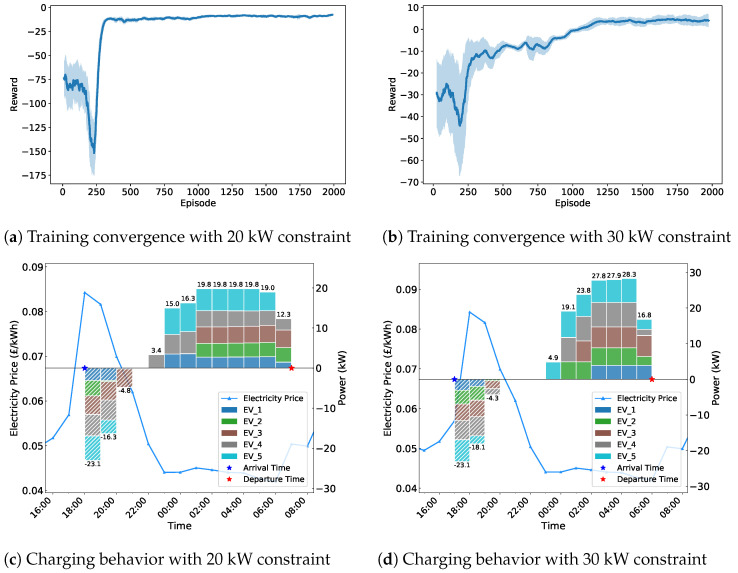
Performance comparison under different power constraints: (**a**,**b**) Training convergence curves showing reward evolution with 20 kW and 30 kW total power limits. (**c**,**d**) Corresponding charging behavior patterns demonstrating how power constraints affect coordination strategies.

Figure 8 illustrates the adaptability of the proposed MADDPG algorithm under different power constraints. The training convergence curves demonstrate that the algorithm successfully converges for both 20 kW and 30 kW total power limits, with similar convergence patterns indicating robust performance across varying constraint scenarios. The charging behavior patterns show that agents effectively adapt their coordination strategies to the imposed power limits. Under the tighter 20 kW constraint, EVs exhibit more conservative charging behaviors with enhanced coordination to avoid exceeding the power limit, while the 30 kW scenario allows for more aggressive charging patterns.

To comprehensively evaluate seasonal robustness, we conduct testing across four disjoint one-month evaluation windows representing different seasons, all selected from periods outside the training dataset. This approach ensures unbiased performance assessment across varying seasonal electricity pricing patterns.

**Figure 9:**
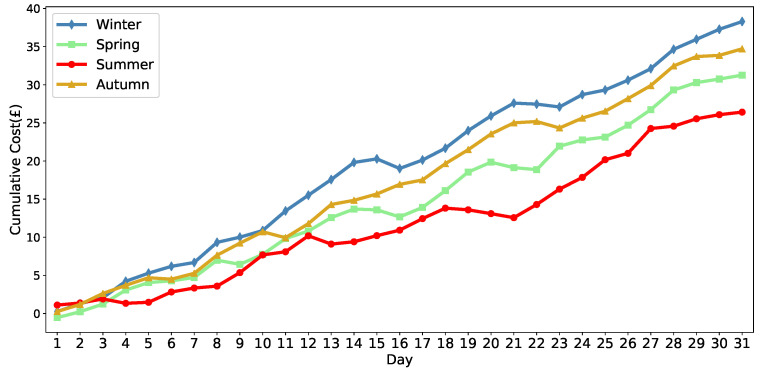
Seasonal cumulative cost performance across four evaluation periods.

Figure 9 demonstrates the cumulative cost performance across four seasonal evaluation periods. The MADDPG algorithm maintains consistent cost optimization performance across all seasonal conditions, demonstrating similar cost accumulation patterns despite varying electricity pricing environments. This validates the robustness of our multi-agent coordination framework to diverse seasonal electricity pricing environments.

To assess the performance of the proposed MADDPG-based coordinated charging scheduling algorithm, we conducted a comprehensive comparative analysis with three baseline methods. The Greedy-based charging scheduling strategy selects actions that maximize immediate reward without considering future consequences or inter-agent coordination. The MAQL-based charging scheduling method employs multi-agent Q-learning with discretized state representation for coordinated vehicle charging decisions. The MADQN-based charging scheduling algorithm implements a multi-agent Deep Q-Network where each agent’s continuous action is discretized to a symmetric set around zero, scaled by its maximum charge/discharge power. All methods operate under identical environmental settings and constraints. The key distinction lies in action space representation: discrete methods are limited to predetermined power levels, while MADDPG utilizes continuous action spaces. All algorithms were evaluated through multiple independent runs to ensure statistical reliability. The performance evaluation was conducted over a 30-day period under dynamic pricing conditions.

**Figure 10:**
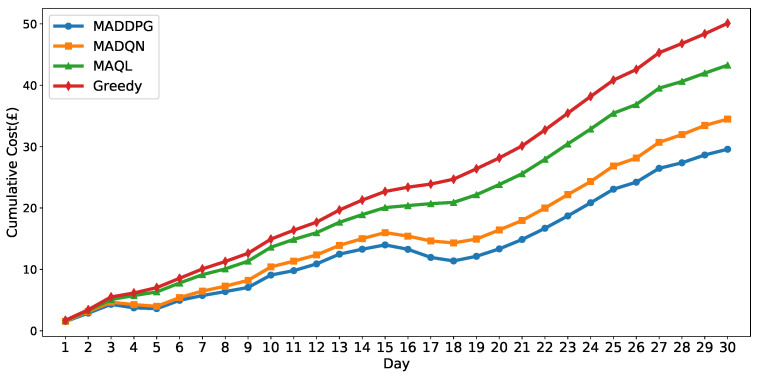
Cumulative charging cost comparison graph for EVs.

Figure 10 and Table 4 present the cumulative charging costs for four scheduling algorithms over a 30-day evaluation period using models trained on six months of historical data. The MADDPG algorithm achieves the lowest cumulative cost with a 41.12% reduction compared with the Greedy baseline, outperforming MADQN by 14.34% and MAQL by 30.91%. While both MADDPG and MADQN show periods of profit generation through V2G operations, MADDPG demonstrates better overall performance than MADQN. These results indicate that the proposed MADDPG method outperforms existing reinforcement learning approaches in this dynamic pricing scenario. For transparency and reproducibility, the detailed per-seed cumulative cost results for all algorithms over 20 independent runs are reported in Appendix A, Table A1.

**Figure 11:**
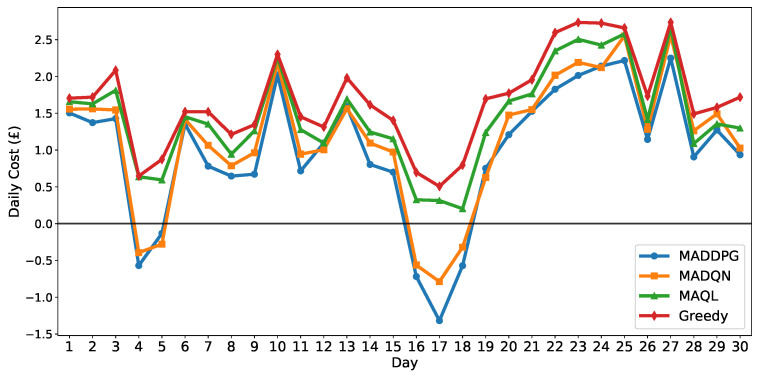
Daily cumulative cost comparison across 30-day evaluation period.

To provide deeper insights into daily cost variations and algorithm reliability, Figure 11 and Table 5 present the daily cost evolution and statistical analysis throughout the evaluation period. Figure 11 illustrates the day-to-day performance characteristics, demonstrating that MADDPG outperforms baseline methods in the majority of daily scenarios with generally lower cost performance. Paired Wilcoxon signed-rank tests reveal that MADDPG achieves *p*-values of $3.7\times 10^{-5}$ and $2.0\times 10^{-6}$, together with Cohen’s *d* values of −1.07 and −1.31 when compared with MADQN and MAQL, respectively. These results demonstrate that the daily cost differences are statistically significant rather than due to random variation, and that the large effect sizes reflect practically meaningful improvements. The corresponding Cliff’s δ values of −0.14 and −0.27 further indicate that MADDPG’s daily costs are generally lower than those of both baseline algorithms. Table 5 provides a comprehensive statistical analysis of daily cost performance, including mean, median, interquartile ranges (IQR), and so on, for all scheduling algorithms. The results confirm that MADDPG achieves the lowest mean daily cost compared with baseline methods.

It is worth noting that our algorithm incorporates battery-protective mechanisms, including SoC constraints and gradual power adjustments through continuous action spaces, which minimize potential lifecycle degradation compared with aggressive charging strategies. Given our evaluation timeframe and these protective measures, battery degradation effects are negligible within our study scope.

## 5. Conclusions

This paper proposed an MADDPG-based coordinated charging scheduling algorithm for multi-agent EV operations in dynamic pricing environments, leveraging smart sensor-enabled IIoT infrastructure within smart grids. The framework employed centralized training with decentralized execution to enable continuous power control, accommodating heterogeneous EV fleets and optimizing charging and discharging decisions in real time. Experimental results show a 41.12% cost reduction over the Greedy baseline, alongside improved grid stability and strong adaptability to electricity price fluctuations and varying demand patterns. Scalability analysis further demonstrated its potential applicability in real-world industrial scenarios under IIoT-enabled smart grids. Future work will extend the framework to consider stochastic external disturbances, physical queuing constraints, and hardware-in-the-loop testing with representative sensors (e.g., smart meters, vehicle charging interface sensors). Evaluation metrics will include the stability of cost savings under varying grid conditions, consistent scheduling performance across scenarios, and the ability to maintain grid voltage and load balance.

## Figures and Tables

**Table 1:** Parameter Settings for EVs’ Behavior.

Parameter	Distribution
Arrival Time (hour)	$N(17,1^{2})$
Departure Time (hour)	$N(6,1^{2})$
Initial SoC	$N(0.3,0.1^{2})$
Target SoC	$N(0.9,0.1^{2})$

**Table 2:** MADDPG Algorithm Parameter Settings.

Parameter	Value
Actor Learning Rate $\alpha_{a}$	$1\times 10^{-3}$
Critic Learning Rate $\alpha_{c}$	$1\times 10^{-3}$
Discount Factor γ	0.98
Target Network Soft Update Rate τ	0.005
Replay Buffer Size |B|	100,000
Minimum Replay Buffer Size ${\left|B\right|}_{\min}$	2000
Batch Size *B*	128
Total Training Episodes *E*	2000

**Table 3:** System Configuration Parameters.

Parameter	Value
Number of EVs	5
EV Battery Capacity Range	25–40 kWh
Max Charging Power per EV	4.0 to 8.0 kW
Total Power Constraint	25.0 kW
Grid Energy Capacity	30 kWh
Time Slot Duration	1 h
SoC Constraints	[0.1, 1.0]

**Table 4:** Accumulated Electricity Purchase Cost of EVs Under Different Scheduling Algorithms.

Scheduling Algorithm	Cumulative Cost (GBP)					
	Day 5	Day 10	Day 15	Day 20	Day 25	Day 30
MADDPG	3.6043	9.0806	13.9893	13.3423	23.0675	29.4552
MADQN	3.9888	10.4051	15.9883	16.4228	26.8537	34.3866
MAQL	6.3302	13.6059	20.0723	23.8202	35.4404	42.6238
Greedy	7.0304	14.9308	22.6946	28.1631	40.8343	50.0182

**Table 5:** Statistics for Daily Costs Under Different Scheduling Algorithms.

Scheduling Algorithm	Daily Cost (£)					
	Mean	Median	IQR (Q1–Q3)	Worst Case	Best Case	Std. Dev.
MADDPG	0.986	1.121	0.704–1.521	2.250	−1.318	0.903
MADQN	1.149	1.272	0.950–1.558	2.560	−0.788	0.886
MAQL	1.442	1.354	1.110–1.746	2.669	0.203	0.670
Greedy	1.670	1.655	1.360–1.974	2.735	0.507	0.626

## Data Availability

The data presented in this study are available on request from the corresponding author. The data are not publicly available due to copyright.

## Appendix A

To enhance transparency and reproducibility, this appendix lists the cumulative charging costs for each of the 20 independent runs across the four scheduling algorithms evaluated in this study. These per-seed results complement the aggregated statistics presented in Section 4.2 and further demonstrate the robustness of the observed performance improvements. The Greedy strategy also exhibits variation across runs due to stochastic initialization of the simulation environment.

**Table A1:** Cumulative charging costs (GBP) for 20 independent runs across all algorithms.

Seed	MADDPG	MADQN	MAQL	Greedy
0	27.8945	32.1456	39.8234	47.2341
5	28.9567	33.4789	41.2567	48.5678
12	30.2341	35.8901	44.7891	51.2345
23	29.1234	33.7856	42.1456	49.1789
25	28.4567	32.8945	40.5678	48.0123
42	31.0789	37.2341	46.5789	52.8901
47	29.7891	34.9567	43.4567	50.3456
52	28.1345	31.8904	39.2341	47.9891
55	30.8901	36.7891	45.9234	53.4567
66	29.4567	34.2345	42.7891	49.8234
78	28.7234	33.1567	40.8901	48.9567
85	30.5678	36.1234	44.3456	51.7891
99	29.2341	33.9567	41.6789	49.5678
101	27.6789	31.5678	38.7891	46.3456
123	31.3456	38.1234	47.8901	54.2345
157	29.8234	35.4567	43.8234	50.7891
178	28.5678	32.7891	40.1234	48.8901
186	30.1234	35.6789	44.5678	51.4567
195	29.6789	34.5678	42.3456	49.9234
225	29.3456	33.0123	41.4567	49.6789
Mean	29.4552	34.3866	42.6238	50.0182
Std. Dev.	1.0496	1.8505	2.5111	2.0544
